# Spectrophotometric Determination of Cefetamet Pivoxil Hydrochloride and Pitavastatin Calcium in Tablet Dosage form

**DOI:** 10.4103/0250-474X.45408

**Published:** 2008

**Authors:** N. H. Vadia, Vandana Patel, H. N. Bhalara

**Affiliations:** Pharmaceutical Quality Assurance Laboratory, Pharmacy Department, Faculty of Technology and Engineering, The Maharaja Sayajirao University of Baroda, Vadodara-390 001, India

**Keywords:** Cefetamet pivoxil hydrochloride, difference spectrophotometry, pharmaceutical formulations, pitavastatin calcium

## Abstract

Two simple, rapid, specific and accurate analytical methods for the estimation of cefetamet pivoxil hydrochloride and pitavastatin calcium in bulk drug and in their tablet formulations are described. These methods are based on difference spectrophotometry, wherein the measurement is done at maximum 221 nm and minimum 275 nm for cefetamet whereas at maximum 240 nm and minimum 259 nm for pitavastatin. The Beer's law was obeyed in the concentration range of 1-35 μg/ml and 1-25 μg/ml and the molar absorptivities were 1.3×10^4^ lit mol^−1^ cm^−1^ and 2.4×10^4^ lit mol^−1^ cm^−1^ for cefetamet pivoxil hydrochloride and pitavastatin calcium, respectively. The proposed methods were validated and successfully applied to the estimation of drugs in tablet formulations.

Cefetamet, 2,2-dimethylproponyloxymethyl(6R,7R)-7-[(2)-2-(aminothiazole-4-yl)-2-methoxyiminoacetylamino]-3-methyl-8-oxo-5-thia-1-azabicyclo[4.2.0]oct-2-ene-2-carboxylate monohydrate (CPH), is an oral third generation cephalosporin antibacterial antibiotic which gets hydrolyzed to form the active agent cefetamet^1^,^2^. Cefetamet, because of its broad spectrum that covers most gram negative and gram positive community acquired pathogens, is one of the drugs of choice in the empiric therapy of respiratory and urinary community acquired infection^3^. Pitavastatin calcium^4^ (PTC), monocalciumbis {(3R,5S,6E)-7-[2-cyclopropyl-4-(4-flurophenyl)-3-quinolyl]-3-5-dihydroxy-6-heptenoate}, is a lipid-lowering agent^5^, used in hyperlipidemia. The analytical methods reported in literature includes HPLC^6^ and polarographic methods^7^ for CPH and LC/MS method^8^ for PTC, however, no spectrophotometric method has so far been reported for these drugs. Hence, it was thought worthwhile to develop advanced spectrophotometric method for the same. This paper describes difference spectrophotometric methods for the estimation of CPH and PTC separately in bulk and their tablet formulations.

A Shimadzu UV-1601 UV/Vis spectrophotometer with 10 mm matched quartz cells was used for all the absorbance measurements. Magnetic stirrer (Remi Equipment Pvt. Ltd., India) was used in the initial steps of extraction. Whatman filter paper No.42 was used to filter the solutions. The CPH and PTC standards were kindly gifted by Alembic Ltd., Vadodara and Zydus Cadila, Ahmedabad, India, respectively. All the chemicals were of analytical reagent grade and solutions were prepared with purified water of IP^9^ grade. Methanol of AR grade was purchased from different suppliers. The solutions of 0.1N HCl and 0.1N NaOH were prepared in water as per IP^9^. Stock solutions of CPH and PTC were prepared in methanol as 1 mg/ml solution. Standard solutions of CPH and PTC were prepared by diluting aliquots (0.1-0.35 ml for CPH and 0.1-0.25 for PTC) of stock solutions to 10 ml with 0.1N NaOH in one set and with 0.1N HCl in other set. The difference spectrum was obtained by treating the acidic form as blank and the basic form as the sample and scanning the spectrum from 200 to 400 nm. The peak maximum was obtained at 221 nm and minimum at 275 nm for CPH and at 240 nm and 259 nm, respectively for PTC (fig. 1). The difference in amplitude at the maximum and minimum were plotted against their respective concentrations and linearity was observed in the concentration range of 1-35 μg/ml for CPH and 1-25 μg/ml for PTC. The optical characteristics such as beer's law limits, molar absorptivities etc. are summarized in Table 1.

**Fig. 1:**
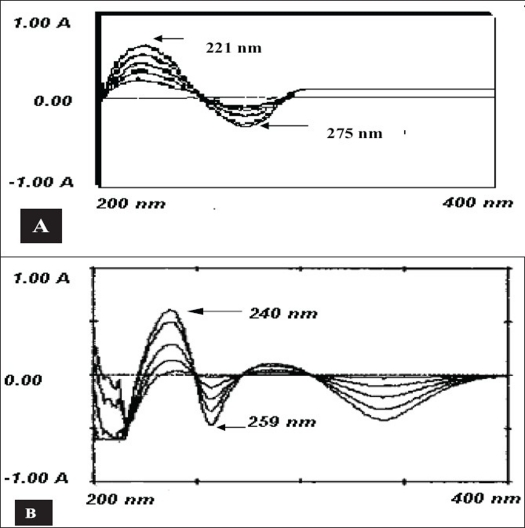
Difference spectrum of cefetamet pivoxil hydrochloride and pitavastatin calcium Difference spectrum of (A) cefetamet pivoxil hydrochloride and (B) pitavastatin calcium. The difference spectrum was obtained by treating the acidic form as blank and the basic form as the sample.

**TABLE 1:** OPTICAL CHARACTERISTICS AND OTHER PARAMETERS FOR CEFETAMET PIVOXIL HYDROCHLORIDE AND PITVASTATIN CALCIUM

Data	Cefetamet Pivoxil Hydrochloride	Pitavastatin Calcium
λ_min_ (nm)	275	259
λ_max_ (nm)	221	240
Beer's law range (μg/ml)	1 to 35	1 to 25
Molar extension coefficient (lit mole^−1^ cm^−1^)	1.3 × 10^4^	2.4 × 10^4^
Sandell's sensitivity μg/cm^2^/0.001 absorbance units	0.03040	0.1827
Regression equation	0.023 × + 0.0036	0.0052 × + 0.0009
Slope	0.023	0.0052
Intercept	0.0036	0.0009
Limit of detection (μg/ml)	0.0876	0.2976
Limit of quantification (μg/ml)	0.2930	0.9923
Correlation coefficient	0.9997	0.9999
Precision (% RSD)	<1%	<2%

Ten tablets each having the strength CPH, 250 mg and 500 mg and PTC, 1 mg and 2 mg, were weighed and ground to a fine powder. A quantity equivalent to 50 mg of the drug was transferred to a 50 ml volumetric flask. It was dissolved in methanol to prepare the stock solution of 1 mg/ml. The solution was filtered through Whatman No. 42 filter paper. Five ml of this solution was then diluted to 50 ml with 0.1N HCl and 0.1N NaOH to get 100 μg/ml solutions in 0.1N HCl in and 0.1N NaOH, separately. Appropriate aliquots were then taken in such a way that the final concentrations in 10 ml volumetric flasks were within the range used for testing the drug by the developed methods. The absorbance of the solutions was measured and the amount of CPH and PTC was computed from the calibration curve. The estimation was repeated six times and the results are given in Table 2.

**TABLE 2:** ANALYSIS OF TABLETS OF CEFETAMET PIVOXIL HYDROCHLORIDE AND PITAVASTATIN CALCIUM

Formulation	Label Claim (mg/tablet)	Amount Found(mg/tablet)	% Label Claim	% RSD*
Cefetamet pivoxil hydrochloride				
Brand 1	250	248.90	99.56	0.3470
Brand 2	500	495.46	99.09	0.4650
Pitavastatin calcium				
Brand 1	1	1.01	101.46	0.2260
Brand 2	2	1.99	99.54	0.2380

* Mean and RSD (relative standard deviation) of six determinations. Tablets were procured from local market

The methods were validated^10^ in terms of accuracy, precision and reproducibility. The Accuracy of method was determined by adding of known quantities of standard drug solution to pre-analyzed sample at three different concentration levels Table 3. Values greater than 99.0% indicate that the proposed methods are accurate for the analysis of drugs. Also, the experiment was repeated three times in a day to determine intra-day precision and on three different days to determine inter-day precision. The percent coefficient of variance (% CV) was calculated at each concentration level. The reproducibility was confirmed by repeating the methods, taking methanol from three different manufacturers and by three different analysts, and the percent relative standard deviation (% RSD) was calculated. The results of method validation are given in Tables 3 and 4.

**TABLE 3:** RECOVERY STUDIES OF CEFETAMET PIVOXIL HYDROCHLORIDE AND PITAVASTATIN CALCIUM

Conc. of formulation (μg/ml)	Std. spiked(μg/ml)	Total Conc. taken (μg/ml)	Total conc. found (μg/ml)	% Recovery*
Cefetamet pivoxil hydrochloride				
10	2	12	11.84	99.05±0.42
10	3	13	12.92	99.46±0.33
10	4	14	14.01	100.12±0.42
30	2	32	32.10	100.32±0.52
30	3	33	32.92	99.76±0.34
30	4	34	33.75	99.28±0.36
Pitavastatin calcium				
10	2	12	12.05	100.48±0.55
10	3	13	12.90	99.26±0.34
10	4	14	13.86	99.03±0.24
15	2	17	16.94	99.66±0.46
15	3	18	17.90	99.46±0.44
15	4	19	18.98	99.29±0.38

* Average±standard deviation of six determinations

**TABLE 4:** PRECISION STUDIES AND REPRODUCIBILITY DATA OF CEFETAMET PIVOXIL HYDROCHLORIDE AND PITAVASTATIN CALCIUM

Performance parameters	Results	

	Cefetamet pivoxil hydrochloride	Pitavastatin calcium
Precision		
Intraday (% CV)*	0.654	0.865
Interday (% CV)*	0.626	0.987
Reproducibility (% RSD)*	0.587	0.879

* Average of six determinations, CV= coefficient of variance and RSD= relative standard deviation

The statistical parameters in method validation studies for accuracy, precision and reproducibility justified the validity of proposed methods. The results of assay and method validation studies given in Tables 2–4 have shown that the methods are simple, accurate, precise, specific and are free from excipient interference.
